# Damage Identification for Underground Structure Based on Frequency Response Function

**DOI:** 10.3390/s18093033

**Published:** 2018-09-10

**Authors:** Shengnan Wang, Xiaohong Long, Hui Luo, Hongping Zhu

**Affiliations:** School of Civil Engineering & Mechanics, Huazhong University of Science and Technology, Wuhan 430074, China; wangsn@hust.edu.cn (S.W.); hpzhu@mail.hust.edu.cn (H.Z.)

**Keywords:** damage identification, frequency response function, model updating, scale model experiment, soil box

## Abstract

Damage identification that is based on modal analysis is widely used in traditional structural damage identification. However, modal analysis is difficult in high damping structures and modal concentrated structures. Unlike approaches based on modal analysis, damage identification based on the frequency response function allows for the avoidance of error and easy verification through other test points. An updating algorithm is devised is this study by utilizing the frequency response function together with the dynamic reduction with respect to the selected design parameters. Numerical results indicate that the method can be used to define multiple parameters with large variation and incomplete measurement data and is robust against measurement noise. With the purpose of avoiding the occurrence of resonance and gaining additional information, the trial and error method has been used to choose a proper frequency. Furthermore, an experimental scale model in a soil box is subjected to the excitation of moving load to validate the effectiveness of the damage identification approach. The improved damage identification method for underground structures, which is based on the analysis of the frequency response function, can be adopted as an efficient and functional damage identification tool.

## 1. Introduction

Structural damage is caused by changes in the material or geometric properties of systems and it is a general problem in the structural design phase and during the operation of structures, especially large and complex civil urban underground infrastructures. Traditionally, the complexity of the underground environment and the degradation failure of materials are the main causes of damage, which affects the service performance of underground structures [1,2,3,4,5].

In previous research, numerous approaches have been developed for identifying damages in structures [6,7,8]. These approaches can be broadly classified into global and local techniques [9,10,11,12]. Dynamic and static measures can be used for damage identification [13,14,15], and damage identification based on modal analysis is the most widely adopted method [16,17,18]. Palaez and Krawczuk [19] proposed a promising damage identification approach that is based on structural vibration. Juan and Hojjat [20] proposed an approach that is based on vibration and enables real-time monitoring. Qu and Chen [21] derived a seismic damage diagnosis method for frame structures while using an artificial neural network (ANN). Yang and Liu [22] proposed a structural damage identification approach with flexibility. Several methods have been proposed due to differences in parameters. The equilateral triangle resonator (ETR) index, which was proposed by Zong and Huang [23], is the most commonly adopted method. Meanwhile, Cha [24] and Moradipour et al. [25] utilized modal strain energy sensitivity for damage identification in civil structures. TaeKim et al. [26] and Feng et al. [27,28] proposed damage detection applications utilizing mode shapes, and some progress has been made. Sensitivity approaches generate the objective function for the first-order Taylor expansion. This approach was used by Yi [29] to analyze the relationship between vibration characteristics and stiffness reduction. Damage identification that is based on statistical information has been proposed [30,31,32,33]. Compared with modal parameters based method, the FRF (Frequency Response Function) based method [34] proposed by some researchers [35,36] has received more attention and applications [37,38,39,40] in recent years due to its advantage, as follows: At first, the measured FRF data can be utilized directly without transformation. In some software, the calculation of modal parameters is based on the measured FRF data. What is more, the modal analysis is more complex, and the error might arise from the modal identification. The identification error might greater than the modelling error. Secondly, the FRF can be measured in more locations of the structure and taken as an objective so it can provide more data. Tranxuan [41] detected the structural damage using measured frequency response function data. Crema and Mastroddi [42] proposed a general procedure based directly on the measured frequency response function data, which can update numerical spatial model or directly to identify and quantify possible damage and failure of the structure. Zou [43] presented an approach for structural damage identification that is based on frequency response function and genetic algorithm (GA). Khoshnoudian and Esfandiari [44] developed a global algorithm for damage assessment of structures based on a parameter estimation method whole using the finite element and measured the modal response of the structure. Yang and Song [45] verified a method of truss damage identification is proposed based on the principal component analysis and frequency response functions. What is more, damage identification based on the frequency response function allows the avoidance of error and easy verification through other test points [46,47]. This technique is commonly used in light structures, mini-type structures, and bridges and is rarely adopted for long-lining underground structures.

This study devised an algorithm that utilizes the frequency response function together with the dynamic reduction with respect to the selected design parameters (Section 2). Section 3 presents an experiment on a scaled aluminum tube model that was conducted to validate the effectiveness of the damage identification approach; the model was used to simulate a long-lining underground structure subjected to the excitation of moving load. Meanwhile, the results of the experiment and simulation were compared theoretically and experimentally. The comparison indicated that the improved damage identification, which was based on the analysis of the frequency response function, for long-lining underground structures could be applied as an efficient and functional damage identification tool.

## 2. Damage Identification Theory based on Acceleration Frequency Response Function

First, parameter estimation expressions are established by the acceleration frequency response function, which is calculated by experimental measurement and model calculation.

The dynamic behavior of a multi-degree-of-freedom structure that is excited by a set of multiforces is described by the following matrix equation:

(1) $$Mx^{\prime \prime }+Cx^{\prime }+Kx=F,$$

where M, C, and K are the mass, damping, and stiffness matrices of the structure, respectively; and x, $x^{\prime }$, and $x^{\prime \prime }$ are the displacement, velocity, and acceleration of the structure, respectively.

By substituting $x=xe^{i\omega t}$ into Equation (1), the function in the frequency domain can be obtained, as follows:

(2) $$-M\omega^{2}xe^{i\omega t}+C\omega ixe^{i\omega t}+Kxe^{i\omega t}=F_{0}\sin\omega t.$$

Frequency response function is the ratio of the input and output of the viscous damping system, which has 3n degrees-of-freedom, with harmonic excitation. The input is the harmonic excitation, and the output is the acceleration response. The frequency response function of structure can be expressed in Equation (2).

(3) $$H(\omega )=\frac{F(\omega )}{X(\omega )},$$

where X(ω) is the steady acceleration response, H(ω) is the acceleration frequency response function, and F(ω) is the harmonic excitation.

With substitution of $x=xe^{i\omega t}$ and Equations (1) into (3), the finite element analysis (FEA) model and the acceleration frequency response function matrix are expressed as Equations (4) and (5), respectively.

(4) $$H_{s}(\omega )={\left[M_{s}-\frac{iC_{s}}{\omega }-\frac{K_{s}}{\omega^{2}}\right]}^{-1},$$

(5) $$H_{e}(\omega )={\left[M_{e}-\frac{iC_{e}}{\omega }-\frac{K_{e}}{\omega^{2}}\right]}^{-1},$$

where $M_{s}$, $C_{s}$, and $K_{s}$ are the mass, damping, and stiffness matrices of the structure by simulation, respectively; $M_{e}$, $C_{e}$, and $K_{e}$ are the mass, damping, and stiffness matrices of the structure by experiment, respectively; and, ω is the excitation frequency.

The damping matrix has only a small effect on the damage and this part can be thus commonly ignored. Equations (4) and (5) can be simplified as Equations (6) and (7), respectively.

(6) $$H_{s}(\omega )={\left[M_{s}-\frac{K_{s}}{\omega^{2}}\right]}^{-1},$$

(7) $$H_{e}(\omega )={\left[M_{e}-\frac{K_{e}}{\omega^{2}}\right]}^{-1}.$$

In the case of a damaged structure, the stiffness matrices after damage according to Equations (6) and (7), is commonly expressed, as follows:

(8) $$\begin{array}{cc} \Delta K & =K_{s}-K_{e}\\ & =\omega^{2}M-\omega^{2}M+K_{s}-K_{e})\\ & =(-\omega^{2}M+K_{s})-(-\omega^{2}M+K_{e})\\ & =\omega^{2}(-H_{s}{\left(\omega \right)}^{-1}+H_{e}{\left(\omega \right)}^{-1}) \end{array},$$

where $K_{s}$ is the stiffness matrices before damage, $K_{e}$ is the stiffness matrices after damage, and ΔK is the variation in the stiffness matrices after damage. Furthermore, the damping matrices are commonly considered constant with the damage that occurs, and the degradation of mass (ΔM) is regarded as zero [48,49,50].

However, in this calculation of $H^{-1}$, a difficult problem is that all of the frequency response function values cannot be measured. Therefore, Equations (6) and (7) can be transformed, as follows:

(9) $$H_{s}(\omega )K_{s}=\omega^{2}H_{s}(\omega )M_{s}-\omega^{2}I,$$

(10) $$H_{e}(\omega )K_{e}=\omega^{2}H_{e}(\omega )M_{e}-\omega^{2}I,$$

where I is identity matrix with 1’s on diagonal line and zeros elsewhere.

Take row x for example, the equation above can be considered, as follows:

(11) $${\left[H_{x1},H_{x2},H_{x3}\ldots \ldots H_{xn}\right]}_{e}{\left(K_{e}\right)}_{n\times n}=\omega^{2}{\left[H_{x1},H_{x2},H_{x3}\ldots \ldots H_{xn}\right]}_{e}{\left(M\right)}_{n\times n}-\omega^{2}[0,0,\ldots ,0,1,0,\ldots ,0,0],$$

where [0,0,…,0,1,0,…,0,0] in Equation (11) is a row matrix with 1’s on the x-th element and zeros elsewhere.

$H_{i}(\omega )$ changes with parameter ω. Therefore, a new matrix can be created only by the first line of every $H_{e}(\omega )$. Equation (11) can be written, as follows:

(12) $$\begin{array}{c} \;{\left[\begin{array}{cc} \begin{array}{cc} H_{11}\left(\omega_{1}\right) & H_{12}\left(\omega_{1}\right)\\ H_{11}\left(\omega_{2}\right) & H_{12}\left(\omega_{2}\right) \end{array} & \begin{array}{cc} \ldots & H_{1n}\left(\omega_{1}\right)\\ \ldots & H_{1n}\left(\omega_{2}\right) \end{array}\\ \begin{array}{cc} \ldots & \ldots \\ H_{11}\left(\omega_{n}\right) & H_{12}\left(\omega_{n}\right) \end{array} & \begin{array}{cc} & \ldots \\ \ldots & H_{1n}\left(\omega_{n}\right) \end{array} \end{array}\right]}_{e}\left[\begin{array}{cc} \begin{array}{cc} K_{11} & K_{12}\\ K_{21} & K_{22} \end{array} & \begin{array}{cc} \ldots & K_{1n}\\ \ldots & K_{2n} \end{array}\\ \begin{array}{cc} \ldots & \ldots \\ K_{n1} & K_{n2} \end{array} & \begin{array}{cc} & \ldots \\ \ldots & K_{nn} \end{array} \end{array}\right]\\ =\left[\begin{array}{cc} \begin{array}{cc} \omega_{1}^{2} & 0\\ 0 & \omega_{2}^{2} \end{array} & \begin{array}{cc} \ldots & 0\\ \ldots & 0 \end{array}\\ \begin{array}{cc} \ldots & \ldots \\ 0 & 0 \end{array} & \begin{array}{cc} & \ldots \\ \ldots & \omega_{n}^{2} \end{array} \end{array}\right]\{{\left[\begin{array}{cc} \begin{array}{cc} H_{11}\left(\omega_{1}\right) & H_{12}\left(\omega_{1}\right)\\ H_{11}\left(\omega_{2}\right) & H_{12}\left(\omega_{2}\right) \end{array} & \begin{array}{cc} \ldots & H_{1n}\left(\omega_{1}\right)\\ \ldots & H_{1n}\left(\omega_{2}\right) \end{array}\\ \begin{array}{cc} \ldots & \ldots \\ H_{11}\left(\omega_{n}\right) & H_{12}\left(\omega_{n}\right) \end{array} & \begin{array}{cc} & \ldots \\ \ldots & H_{1n}\left(\omega_{n}\right) \end{array} \end{array}\right]}_{e}\left[\begin{array}{cc} \begin{array}{cc} M_{11} & M_{12}\\ M_{21} & M_{22} \end{array} & \begin{array}{cc} \ldots & M_{1n}\\ \ldots & M_{2n} \end{array}\\ \begin{array}{cc} \ldots & \ldots \\ M_{n1} & M_{n2} \end{array} & \begin{array}{cc} & \ldots \\ \ldots & M_{nn} \end{array} \end{array}\right]-\left[\begin{array}{cc} \begin{array}{cc} 1 & 0\\ 1 & 0 \end{array} & \begin{array}{cc} \ldots & 0\\ \ldots & 0 \end{array}\\ \begin{array}{cc} \ldots & \ldots \\ 1 & 0 \end{array} & \begin{array}{cc} & \ldots \\ \ldots & 0 \end{array} \end{array}\right]\} \end{array}.$$

Due to the requirement of calculation in this paper, the first two rows and two columns $\left[\begin{array}{cc} K_{11} & K_{12}\\ K_{21} & K_{22} \end{array}\right]$ of stiffness matrix $\left[\begin{array}{cc} \begin{array}{cc} K_{11} & K_{12}\\ K_{21} & K_{22} \end{array} & \begin{array}{cc} \ldots & K_{1n}\\ \ldots & K_{2n} \end{array}\\ \begin{array}{cc} \ldots & \ldots \\ K_{n1} & K_{n2} \end{array} & \begin{array}{cc} & \ldots \\ \ldots & K_{nn} \end{array} \end{array}\right]$ are extracted in Equation (12). Moreover, because the symmetry of the simplified stiffness matrix, the number of unknown parameters can be reduced, $\left[\begin{array}{c} K_{11}\\ K_{21} \end{array}\right]$ was adopted. Therefore, Equation (12) can be simplified into Equation (13).

(13) $$\left[\begin{array}{c} \begin{array}{c} \begin{array}{cc} H_{12}\left(\omega_{1}\right) & H_{12}\left(\omega_{1}\right) \end{array}\\ \begin{array}{cc} H_{11}\left(\omega_{2}\right) & H_{12}\left(\omega_{2}\right) \end{array} \end{array}\\ \begin{array}{cc} H_{11}\left(\omega_{3}\right) & H_{12}\left(\omega_{3}\right) \end{array}\\ \begin{array}{c} \begin{array}{cc} \ldots & \ldots \end{array}\\ \begin{array}{cc} H_{11}\left(\omega_{n}\right) & H_{12}\left(\omega_{n}\right) \end{array} \end{array} \end{array}\right]\left[\begin{array}{c} K_{11}\\ K_{21} \end{array}\right]=\left[\begin{array}{c} \begin{array}{c} \omega_{1}^{2}H_{11}\left(\omega_{1}\right)M_{11}-\omega_{1}^{2}\\ \omega_{2}^{2}H_{11}\left(\omega_{2}\right)M_{21}-\omega_{2}^{2} \end{array}\\ \omega_{3}^{2}H_{11}\left(\omega_{3}\right)M_{31}-\omega_{3}^{2}\\ \begin{array}{c} \ldots \\ \omega_{n}^{2}H_{11}\left(\omega_{n}\right)M_{n1}-\omega_{n}^{2} \end{array} \end{array}\right].$$

However, several of the values of $H_{i}(\omega )$ are nearly the same due to the same size and material of the element in practice, thereby possibly affecting damage identification. To avoid this phenomenon, Equation (8) can be expressed, as follows:

(14) $$H_{e}(\omega )\Delta K=\omega^{2}(-H_{e}(\omega )H_{s}{\left(\omega \right)}^{-1}+I).$$

$H_{s}{\left(\omega \right)}^{-1}$ can be calculated using Equation (6). Similarly, a new matrix can be created from the first row of every $H_{e}(\omega )$. Equation (14) can be written, as follows:

(15) $$\begin{array}{c} {\left[H_{11}\left(\omega_{1}\right)\;H_{12}\left(\omega_{1}\right)\;\ldots \;H_{1n}\left(\omega_{1}\right)\right]}_{e}\left[\begin{array}{cc} \begin{array}{cc} \Delta K_{11} & \Delta K_{12}\\ \Delta K_{21} & \Delta K_{22} \end{array} & \begin{array}{cc} \ldots & \Delta K_{1n}\\ \ldots & \Delta K_{2n} \end{array}\\ \begin{array}{cc} \ldots & \ldots \\ \Delta K_{n1} & \Delta K_{n2} \end{array} & \begin{array}{cc} & \ldots \\ \ldots & \Delta K_{nn} \end{array} \end{array}\right]\\ =\omega^{2}\{\left[1\;0\;\ldots \;0\right]-{\left[H_{11}\left(\omega_{1}\right)\;H_{12}\left(\omega_{1}\right)\;\ldots \;H_{1n}\left(\omega_{1}\right)\right]}_{e}{\left[\begin{array}{cc} \begin{array}{cc} H_{11} & H_{12}\\ H_{21} & H_{22} \end{array} & \begin{array}{cc} \ldots & H_{1n}\\ \ldots & H_{2n} \end{array}\\ \begin{array}{cc} \ldots & \ldots \\ H_{n1} & H_{n2} \end{array} & \begin{array}{cc} & \ldots \\ \ldots & H_{nn} \end{array} \end{array}\right]}_{s}\} \end{array}.$$

The degradation of stiffness (ΔK) that is caused by damage is commonly simulated by ΔEI in actual projects [51].

Therefore, Equation (11) can be written, as follows:

(16) $${\left[H_{x1},H_{x2},H_{x3}\ldots \ldots H_{xn}\right]}_{e}(K^{s})\times \Delta \left(EI\right)=\omega^{2}{\left[H_{x1},H_{x2},H_{x3}\ldots \ldots H_{xn}\right]}_{e}{\left({\left[H\right]}_{s}\right)}^{-1}-\omega^{2}[0,0\ldots 1,0\ldots 0,0],$$

where ${\left({\left[H\right]}_{s}\right)}^{-1}=M_{s}-\frac{K_{s}}{\omega^{2}}$. where $\left[K^{s}\right]$ is the stiffness matrix calculated by the finite element model and is a known quantity.

**Figure 1:**
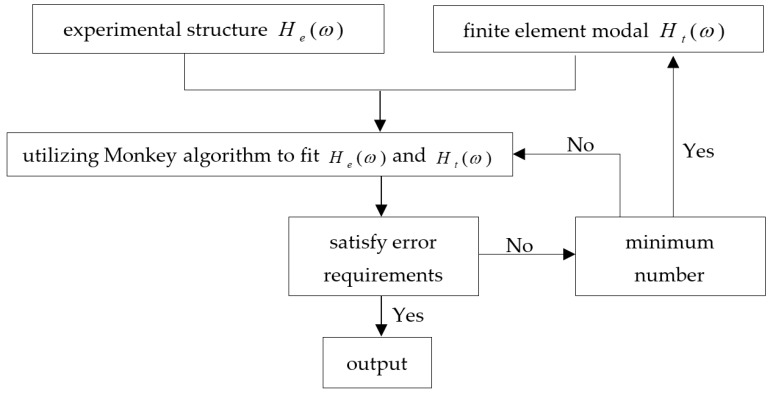
Process of damage identification based on frequency response function.

ΔK can be calculated after the appearance of Δ(EI). Therefore, the location of the damage can be easily evaluated. In this paper, the Monkey algorithm was utilized as a optimization algorithm to fit $H_{e}(\omega )$ and $H_{t}(\omega )$. Monkey Algorithm (MA) is an intelligent optimization algorithm, which is put forward by Zhao and Tang [52], is used to solve multivariable optimization and multimodal function optimization problem. In recent years, many scientists put into the related study of Monkey Algorithm. The establishment of a gas filling station model is shown in the work by Zhao [53], and an intrusion detection technology is worked by Zhang and Sun [54]. Yi has studied on sensor placement on Canton Tower for health monitoring using asynchronous-climb monkey algorithm [55]. What is more, to overcome the limitations overcome the limitations of the monkey algorithm, a reprogram monkey algorithm program is improved by Wang [56]. The algorithm is been inspired by the monkey climbing process in a given field that has many mountains, which simulated by the climbing, watching, and jumping of the monkey in order to find the highest mountaintop. The corresponding search process is as followed: climbing is used to search the local optimal solution of the current location; watching is used to search a better solution of the adjacent of the current location; and, jumping is used to reach a new location which can avoid the local optimum. This algorithm is a process of constantly looking for the optimal solution. Monkey Algorithm was used to identify the change in stiffness, which the error requirements is 0.1% in this paper. Meanwhile, assume that the group number of monkey is 5, and the number of each group is 4. Analysis at the situation when the step size defaults to 1. The process of damage identification that is based on the acceleration frequency response function is shown in Figure 1.

## 3. Experimental Study

### 3.1. Similarity Analysis

Knowledge of similitude rules is essential for satisfying the fundamental conditions in planning the scaled shield tunnel model testing, because the scaled model must behave in a manner similar to the prototype.

Models in previous studies fall into two categories of vibration research. The first category considers the entire vibrating structure a rigid body. In the second group, the model in which vibrations occur in the structure is with respect to its stiffness. The vibration of a scaled shield tunnel model in a soil box with damage belongs to the second research category.

The scaled shield tunnel model in this study is a nonlinear elastic model because it involves the shield tunnel and soil structures. Three requirements must be satisfied simultaneously in designing the scaled shield tunnel model.

(1) Geometry
(2) Mass and mass distribution
(3) Gravity and inertial forces

With the scaled shield tunnel model structure considered to be an infinite Timoshenko beam, the deformation equation is expressed, as follows:

(17) $$\frac{1}{r}=\frac{M}{EI},$$

where r is the radius of curvature, E is Young’s modulus, and I is the moment of inertia.

(18) $$\frac{r_{p}}{r_{m}}=\frac{M_{m}}{M_{p}}\cdot \frac{E_{p}}{E_{m}}\cdot \frac{I_{p}}{I_{m}},$$

where subscripts m and p indicate the model and the prototype, respectively. According to Froude’s scaling law [57], Equation (18) can be simplified to the following:

(19) $$\frac{I_{m}}{I_{p}}={\left(\frac{L_{m}}{L_{p}}\right)}^{5}\cdot \frac{E_{p}}{E_{m}},$$

with $I\propto L^{4}$, where $L_{m}$ and $L_{p}$ are the characteristic length of the model and the prototype, respectively. Equation (19) can be written, as follows:

(20) $$\frac{L_{p}}{L_{m}}=\frac{E_{p}}{E_{m}}.$$

The ratio of the similarity of the shield tunnel structural and soil inertia forces, which are indicated by $F_{ss}$ and $F_{s}$, must be the same in the model and the prototype. The ratio of the structural inertia force $F_{ss}$ can be expressed, as follows:

(21) $$F_{ss}=ma,$$

where m is the mass and a is the acceleration. Thus,

(22) $$F_{ss}\propto \frac{\rho_{ss}L^{4}}{t^{2}},$$

where $\rho_{ss}$ is the shield tunnel structure density and t is the characteristic time. t is directly proportional to $\frac{L}{t}$ and thus can be written as follows:

(23) $$F_{ss}\propto \rho_{ss}L^{2}v^{2},$$

where v is the velocity of moving load.

The requirement for similarity is thus as follows:

(24) $$\frac{F_{ss}}{F_{s}}\propto \frac{\rho_{ss}L^{2}v^{2}}{\rho_{s}L^{2}v^{2}}=\mathrm{constant},$$

where $\rho_{s}$ is the soil density.

Meanwhile, moving load scaling should also be considered in this study because it is the source of the main excitation that acts on the shield tunnel model. The scaling law for the situation in this work is also considered the Froude scaling law, which dictates that the scales for the time and velocity were equal to the square root of the length scale. All of the parameters for the shield tunnel model are shown in Table 1. However, using the same scale for the entire model will result in an excessively small model and hence a conflict with the scale of the density. Similarly, the scale of density may be remarkably small, thereby potentially causing the instability between the shield model and the soil. To avoid these problems, aluminum model pipes are used in this experiment, and their properties are listed in Table 1. The stiffness of the shield tunnel model and the soil box parameters are also shown in Table 1. The sand was considered a part of the shield tunnel system in the soil box. Fine yellow sand is used in the tests for this study.

### 3.2. Description of Tested Structures

The proposed damage identification procedure was verified with real measurement data from moving load vibration tests. Shield tunnel structures are affected by components in the complex environment, such as the surrounding soil and groundwater. Thus, the experimental tests were conducted with a simply scaled aluminum pipe model. The model, which was subjected to the excitation of moving load with all of the designed sensor arrangement scenarios, was placed in the soil box for the segment of shield tunnel for structural damage identification analysis.

**Figure 2:**
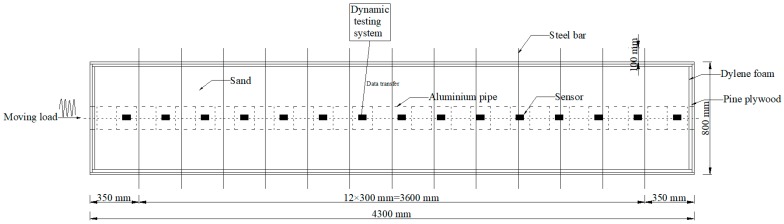
Experimental setup for dynamic tests of aluminum pipe model.

**Figure 3:**
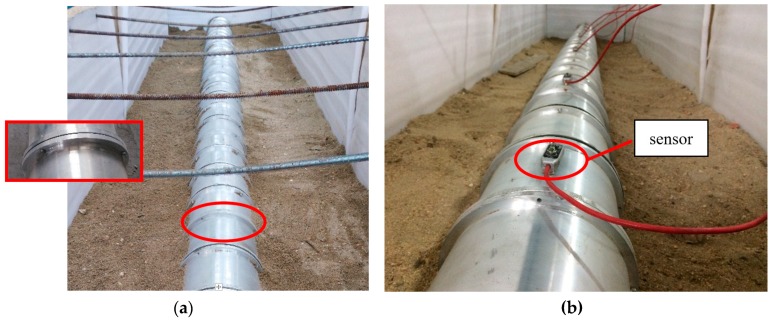
Sketch of aluminum pipe model: (**a**) segments are tighten by the bolts against the flange; (**b**) sensor.

**Figure 4:**
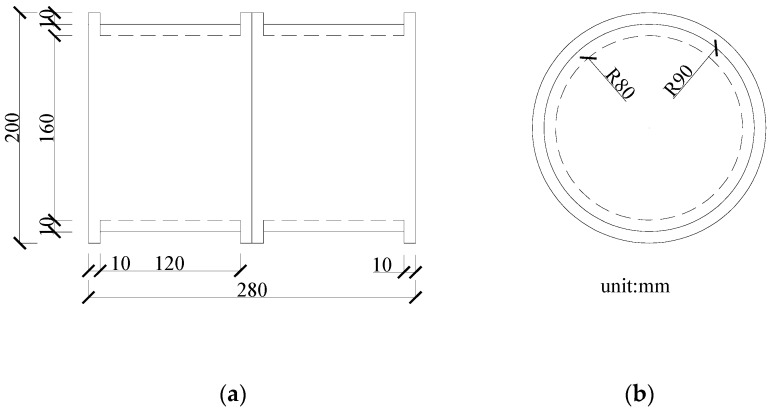
Shape and size information of aluminum segment model (unit: mm): (**a**) side view; (**b**) front view.

Although the tests were not performed in a soil environment, the testing data provided information that demonstrated the reliability of the proposed damage index in detecting damage. In this experiment, aluminum and yellow sand were selected to represent concrete and soil, respectively. The experimental setup for the dynamic tests of the aluminum pipe model is shown in Figure 2. Eighteen cases for the scaled shield tunnel model with different damage types were tested and monitored by using acceleration sensors, as shown in Figure 3. During testing, the entire structure in the model vibrated as a rigid body. Each segment with a flange structure that formed the entire tunnel structure measured approximately 140 mm in length, 180 mm in outer diameter, and 160 mm in inner diameter. The segments installed were tightened by bolts to seal the gasket and washer against the flange, as depicted in Figure 3. Detailed size information about the experimental model is shown in Figure 4. In the model design, each segment is equivalent to a circle, which has a stiffness reduction according to the area and the moment of inertia.

**Figure 5:**
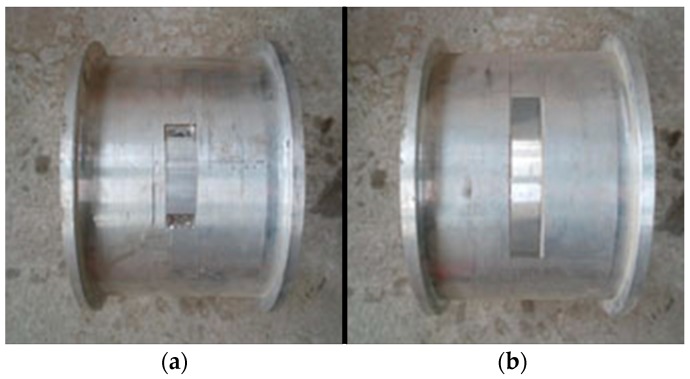
Bar damage scenarios: (**a**) 5% damage; (**b**) 10% damage.

**Figure 6:**
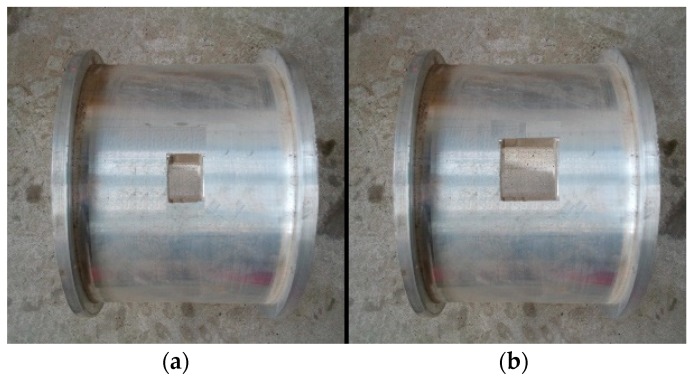
Block damage scenarios: (**a**) 5% damage; and, (**b**) 10% damage.

Two damage types were involved in the study, namely, bar and block damages. Varying damage severities were applied to Groups A and B, which were used to simulate crack and peeling-off damages, respectively. Each damage was located at the top of the aluminum pipe. In Group A, the bending rigidity of the bar damage components was reduced by 5% and 10% (The size of the four damages are 120 mm×20 mm, 240 mm×20 mm), as shown in Figure 5. The same was performed on Group B (The size of the four damages are 50 mm×50 mm, 70 mm×70 mm), as shown in Figure 6. Nine cases were considered for each group. An undamaged case, four single-damage cases, three double-damage cases, and one triple-damage case were set. The details of the two groups are tabulated in Table 2 and Table 3.

**Figure 7:**
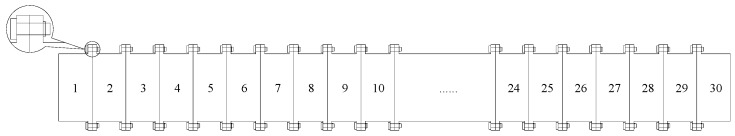
Number of aluminum pipes.

In the experiment, 30 aluminum segments were used to assemble the long-lining shield tunnel structure whose total length was 4200 mm, as shown in Figure 7. Meanwhile, the wireless test system STS LIVE (Bridge Diagnostics, Inc, Louisville, CO, USA) was used to measure and collect the real-time accelerator signals of each sensor. The specifications of the wireless accelerometer are shown in Table 3. Fifteen wireless sensors were evenly distributed on the pipe model.

**Figure 8:**
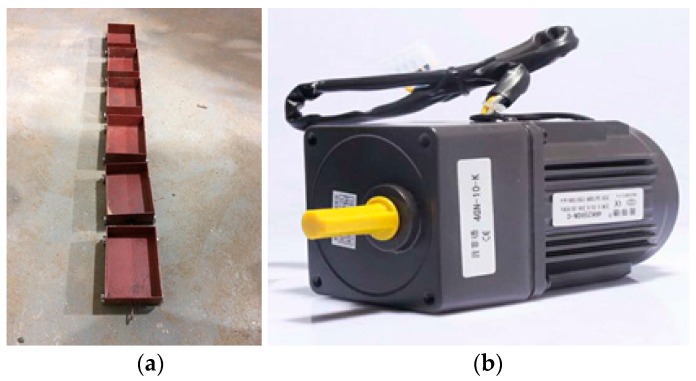
Simulated train load: (**a**) moving train; (**b**) traction device.

A moving load was used to simulate the train load in the tunnel. The simulated moving load consisted of six carriages, and each carriage had two axes with a spacing of 100 mm. The traction device, which could provide stable power, was used to pull the train load travelling in the tunnel. The power of the traction device was 25 watts, which was rotated at 125 revolutions per minute. The moving load and traction device are shown in Figure 8. The sampling frequency was 1000 Hz. “Same measurements” were taken 10 times in order to avoid mistakes and accidental errors. The number of aluminum pipes is shown in Figure 7.

**Figure 9:**
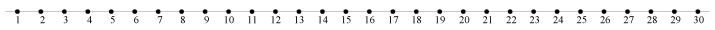
Simplified finite element model.

According to the testing condition, the structure was simplified to a beam, which meant that the response of all the nodes at the same cross section were the same. The finite element model of the test was simplified as a beam structure with 30 elements, as shown in Figure 9. In addition, the experiment was conducted within a short time to ensure accuracy. Temperature and humidity were relatively stable, and no interference was caused by external excitation sources.

### 3.3. Experimental Results and Analysis

Real-time acceleration data were obtained by the wireless node and a wireless test system. To limit the length of this paper, only parts of the results collected by the typical acceleration sensor are shown here.

**Figure 10:**
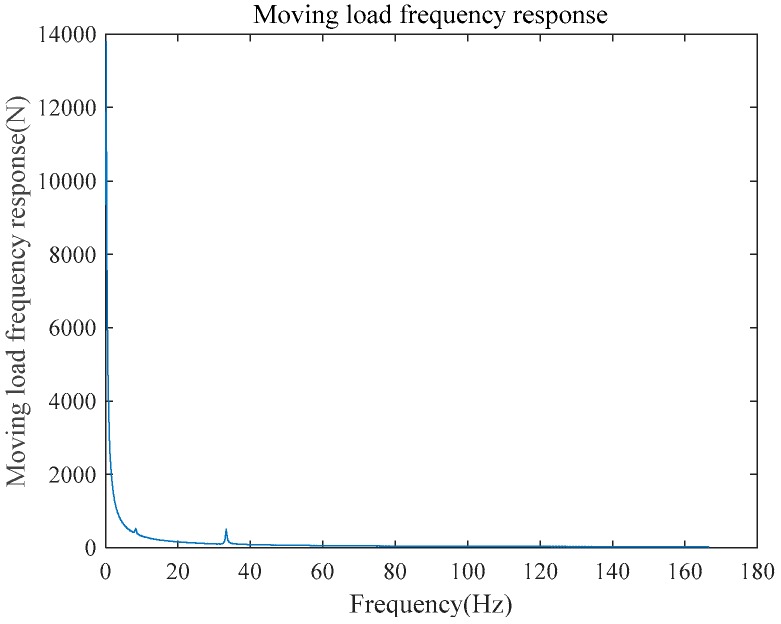
Moving load frequency response.

First, the frequency response function of the undamaged structure was tested. In the experimental tests, the speed of the moving load is 2 m/s. The scaling law for the situation in this work is also considered the Froude scaling law, which dictates that the scales for the time and velocity should be equal to the square root of the length scale. According to the reference [39], equivalent node load can be expressed, as follows:

(25) $$P\left(x,\omega \right)=\frac{F}{\Delta T}e^{-s\frac{-v+\sqrt{v^{2}+2ax}}{a}},$$

where P(x,ω) is the moving load in frequency domain; s is the Laplace parameter; F is the moving load. The frequency response can be obtained with P(x,ω). The frequency response of the simulated moving load, which consisted of six carriages, is shown in Figure 10.

**Figure 11:**
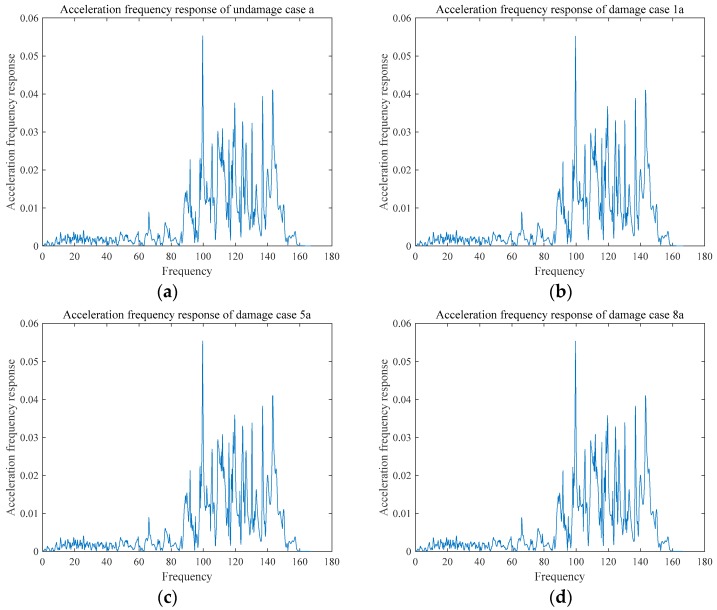
Acceleration frequency response of Group A: (**a**) UDMa; (**b**) DMG1a; (**c**) DMG5a; and, (**d**) DMG8a.

**Figure 12:**
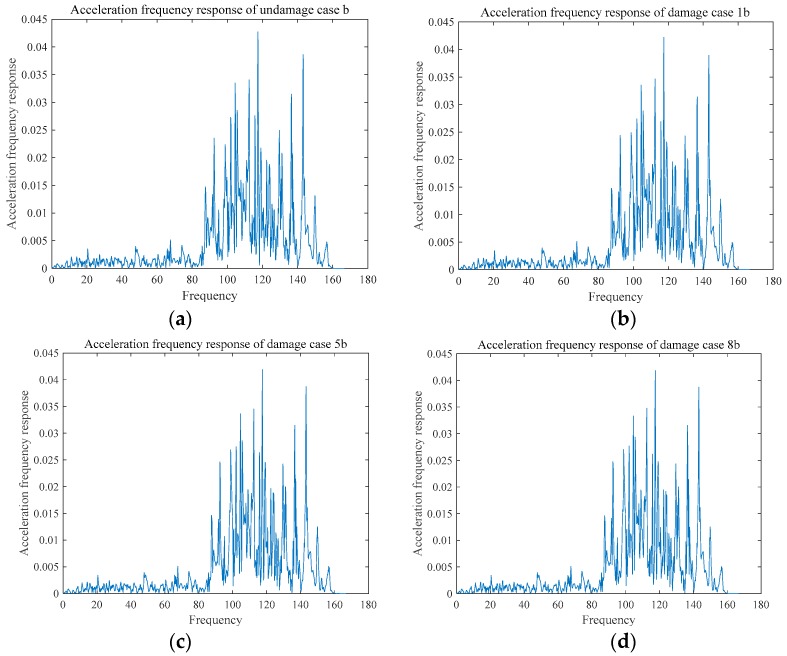
Acceleration frequency response of Group B. (**a**) UDMb; (**c**) DMG5b; and, (**d**) DMG8b.

**Figure 13:**
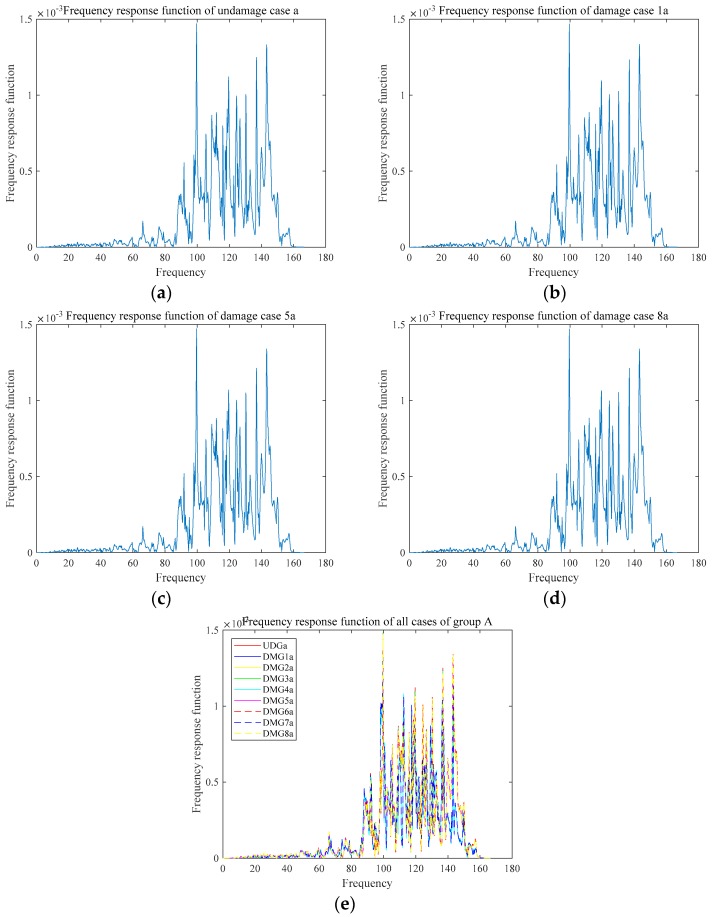
Frequency response function of Group A: (**a**) UDMa; (**b**) DMG1a; (**c**) DMG5a; (**d**) DMG8a; and, (**e**) all cases.

**Figure 14:**
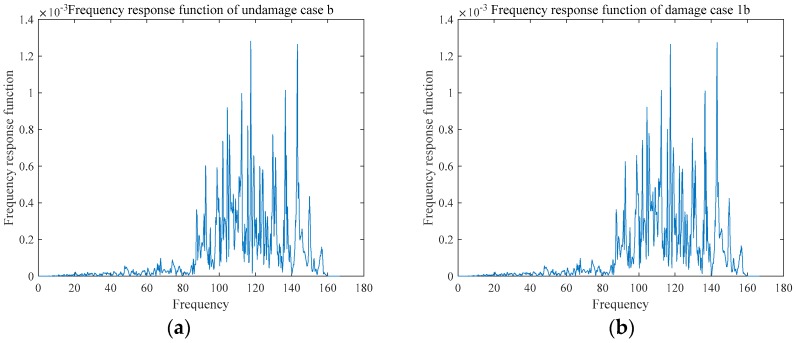

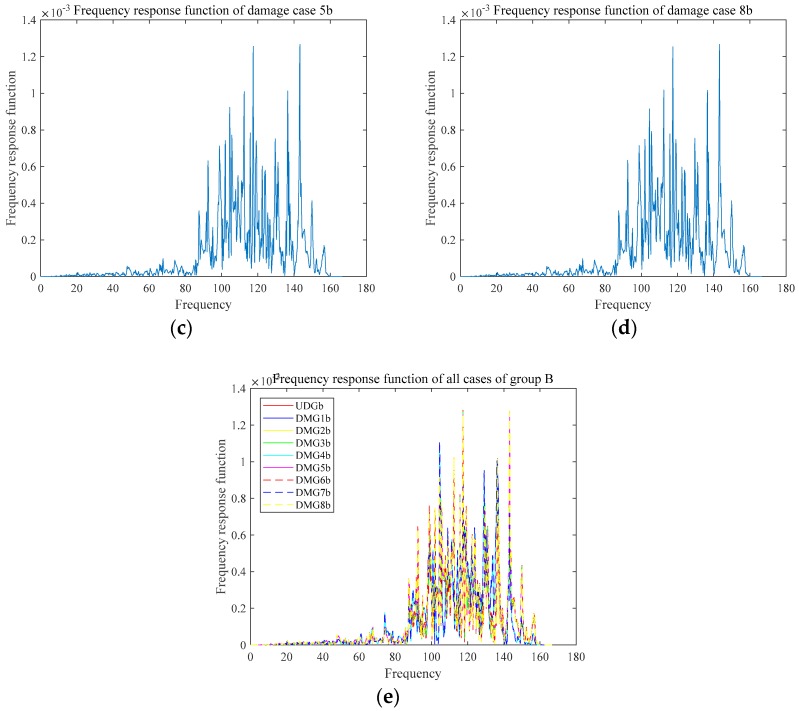
Frequency response function of Group B: (**a**) UDMb; (**b**) DMG1b; (**c**) DMG5b; (**d**) DMG8b; and, (**e**) all cases.

The acceleration frequency responses of the two damages are shown in the Figure 11 and Figure 12, and the frequency response function of the two groups are shown in Figure 13 and Figure 14. Due to limitation of pages, only some representative cases are presented in Figure 11, Figure 12, Figure 13 and Figure 14.

The results of the frequency response function are shown in Figure 13 and Figure 14. Undamaged cases a and b had the same situation. Model updating was used as described in Section 2. To limit the length of the paper, only parts of the results are shown here. The data of the undamaged case (which can be considered the simulation model) and damage case 1a are depicted here to illustrate the effect of damage identification. The modified EI values of the undamaged case and damaged degree of case 1a are presented in Table 4 and Table 5, respectively. Under the undamaged circumstances, the model had been modified, which is shown as Table 4. By using the modified EI as a benchmark of undamaged structure, the experimental data which can obtain damaged $EI_{d}$ was adopted to identify damage which shown as Table 5. In particular, ΔEIEIu is the percentage of damage degree.

**Figure 15:**
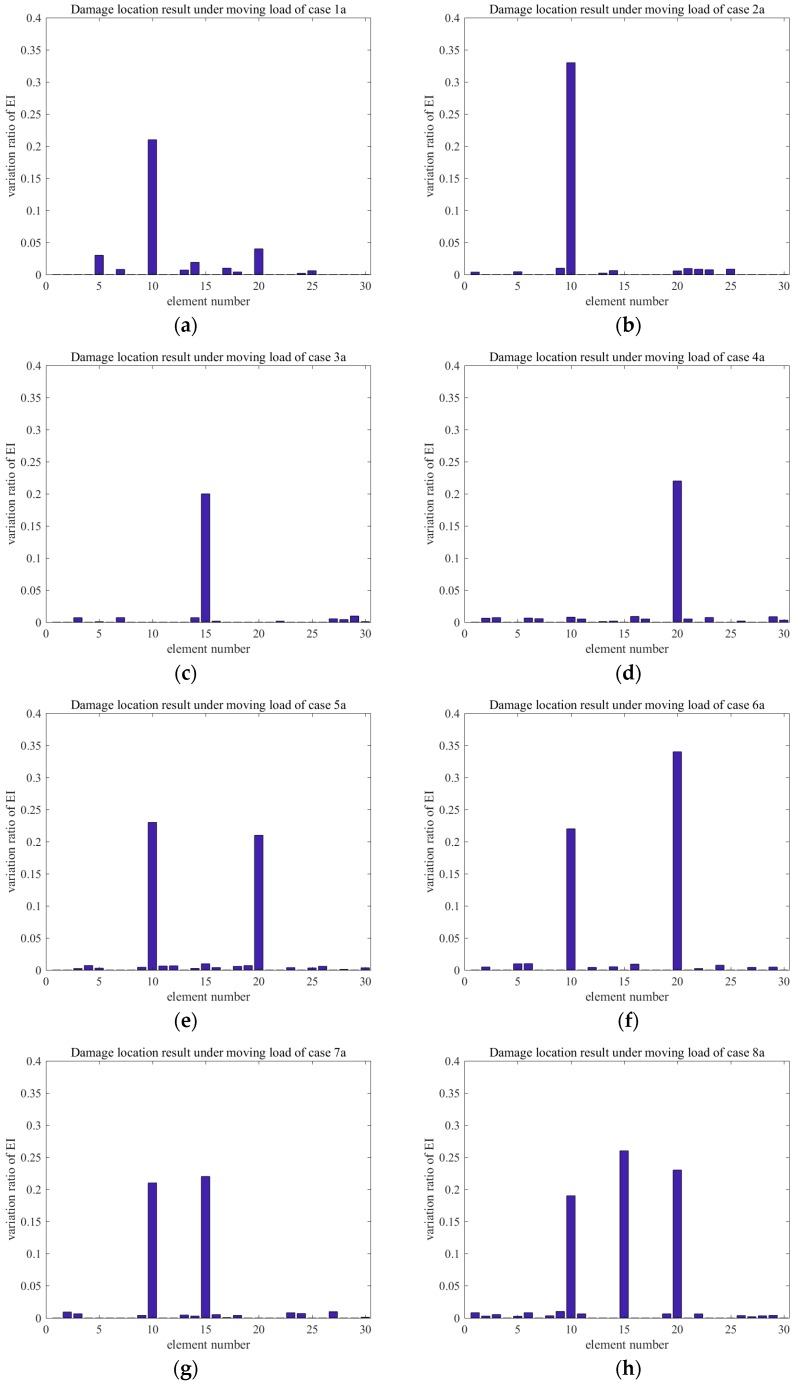
Damage location results under moving load of Group A: (**a**) DMG1a; (**b**) DMG2a; (**c**) DMG3a; (**d**) DMG4a; (**e**) DMG5a; (**f**) DMG6a; (**g**) DMG7a; and, (**h**) DMG8a.

**Figure 16:**
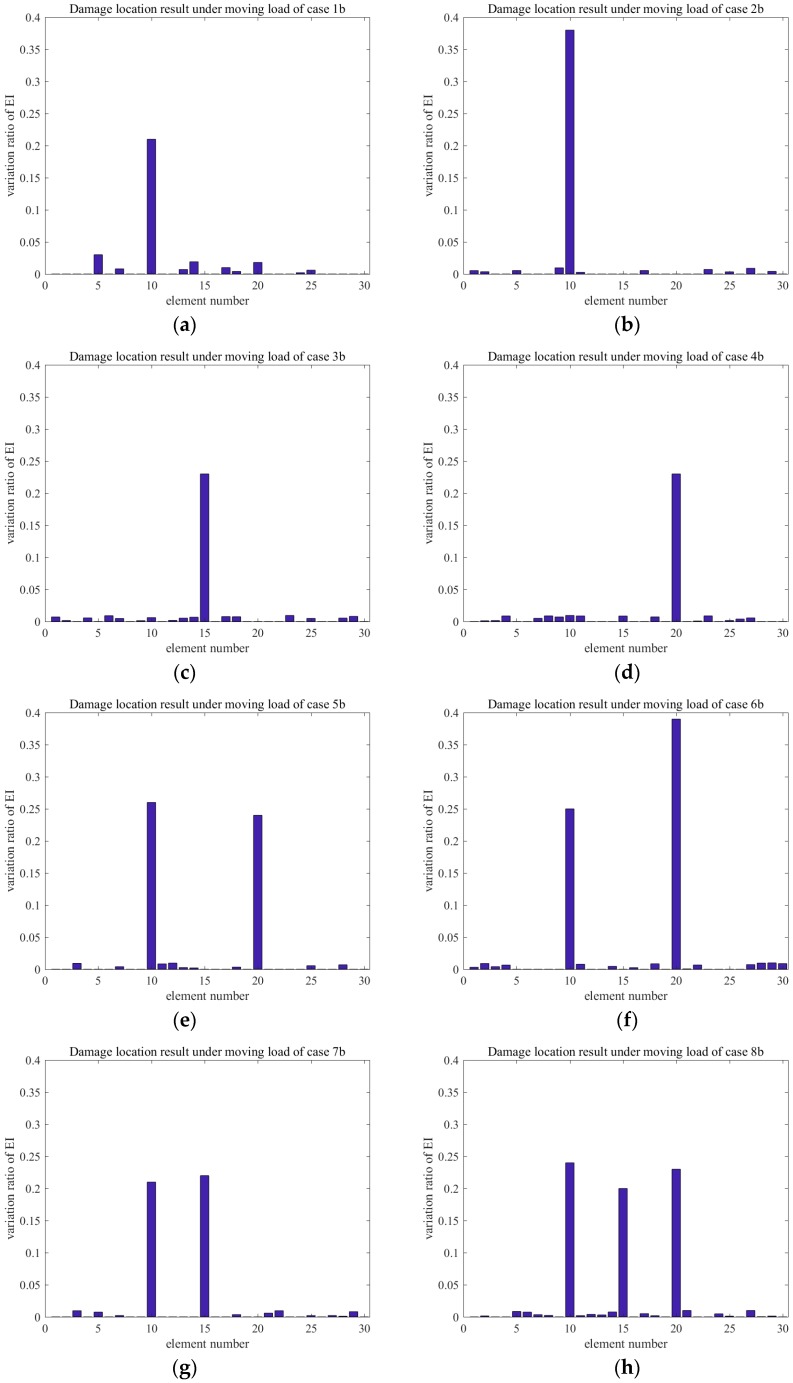
Damage location results under moving load of Group B: (**a**) DMG1b; (**b**) DMG2b; (**c**) DMG3b; (**d**) DMG4b; (**e**) DMG5b; (**f**) DMG6b; (**g**) DMG7b; and, (**h**) DMG8b.

The variation ratios of EI obtained for each damage case of Groups A and B are shown in Figure 15 and Figure 16, respectively. Application of the proposed method revealed that the variation ratio of EI (ΔEI) of the damaged element showed a noticeably increasing trend. The degradation of stiffness that is caused by damage is commonly simulated by ΔEI in actual projects. The testing result indicated that the numerical model was correct. The variation ratios of EI were observed exactly at the damage element locations in single- and multi-damage situations. Furthermore, the bending rigidity of element 10 in case 1a was reduced by 5%. For example, in Group A, the bending rigidity of element 10 was reduced by 10% in case 2a. Meanwhile, the bending rigidity values of elements 15 and 20 were reduced by 5% in cases 3a and 4a, respectively, and a 5% measurement noise was considered in all of the cases. Figure 15 and Figure 16 indicate that the variation ratios of EI at the damaged element were amplified obviously. For example, in case 1a, the variation ratio of EI of the damaged element was 0.21, which largely outweighed those of the other elements. This finding demonstrated the feasibility and accuracy of using the proposed approach in identifying the damage locations in a single-damage situation. Moreover, the variation ratio may also be used for quantifying the damage. In multi-damage situations, the variation ratio tended to increase in comparison with that in the single-damage case. This result may be attributed to the interaction between damages. A comparison of cases 5a and 7a showed that the shorter the distance between the two damage locations, the greater the influence.

**Figure 17:**
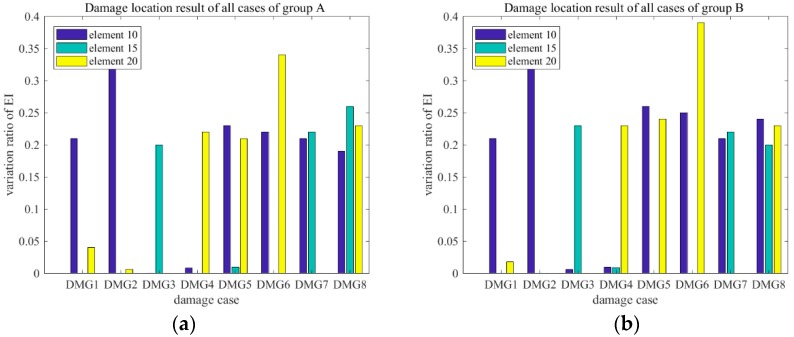
Damage location results of all cases: (**a**) Group A; and, (**b**) Group B.

The damage detection results for elements 10, 15, and 20 under different damage cases are shown in Figure 17. As Figure 17a shows, the variation ratios of EI of the damaged elements were significantly larger than those in the undamaged case. This finding indicated the effective detection of the introduced damage. In case 6a, elements 10 and 20 had damage severities of 5% and 10%, respectively, and element 15 had no damage, as shown in Figure 17a. The damage index of element 15 showed no noticeable change in comparison with that in the undamaged case. However, the damage index values of damaged elements 10 and 20 showed significant increases when compared with those in the undamaged state. A comparison of cases 5a and 6a showed that the damage magnitude has only a small influence on the effect of damage identification. Meanwhile, a comparison of cases 5a and 7a indicated that the shorter the distance between two damage locations, the greater the influence. Figure 17b shows the situation of Group B. The proposed approach was appropriate for detecting the damage severity in the single- and multi-damage situations. This method is thus attractive for detecting scouring damage in shield tunnels because various uncertainties and noises are involved in the modeling of shield tunnel structures and measuring their dynamic responses. However, the quantitative relation between the simulated damage and the actual damage is unclear and it should be discussed further.

## 4. Discussion and Conclusions

This work investigated the feasibility and effectiveness of using the acceleration frequency function to identify the structural damage in underground tunnel structures. Numerical and experimental studies were conducted. An aluminum pipe model with a simulated underground boundary condition was designed and tested in a large soil box. Different damage scenarios were simulated by cutting the pipe models into various damage sizes. The experimental studies performed covered the accelerated frequency response function used for damage identification in underground structures. The stiffness loss ΔK could be calculated after the appearance of Δ(EI). Therefore, the location of the damage could be easily judged. Meanwhile, the damage magnitude had less influence on the effect of damage identification. Moreover, the shorter the distance between two damage locations, the greater the influence. Damage identification for underground structures based on the analysis of the frequency response function can be adopted as an efficient and functional damage identification tool in practical applications. However, the quantitative relation between the simulated damage and the actual damage should be discussed further.

## Figures and Tables

**Table 1:** The properties of aluminum model pipes considered in experimental verification.

	Item	Scale	Prototype	Desired Model	Adopted Model
Soil model	Length	130	135 m	4500 mm	4300 mm
	Height	130	30 m	1000 mm	800 mm
	Depth	130	30 m	1000 mm	800 mm
	Bulk density	11	${1800\;\mathrm{kg}/m}^{3}$	${1800\;\mathrm{kg}/m}^{3}$	${1800\;\mathrm{kg}/m}^{3}$
Shield tunnel model	Inner diameter	130	5.4 m	180 mm	160 mm
	Outer diameter	130	6 m	200 mm	180 mm
	Length	130	135 m	4500 mm	4300 mm

**Table 2:** Damage cases of Groups A and B considered in experimental verification.

Label	Segment No.	Damage Location	Group A		Group B		Damage Severity
			Damage Scenario	Damage Type	Damage Scenario	Damage Type	
**0**	**Null**	**Null**	**UDMa**	**Bar**	**UDMb**	**Block**	**Null**
1	S10	*x* = 9 m~10 m	DMG1a	bar	DMG1b	block	5%
2	S10	*x* = 9 m~10 m	DMG2a	bar	DMG2b	block	10%
3	S15	*x* = 14 m~15 m	DMG3a	bar	DMG3b	block	5%
4	S20	*x* = 19 m~20 m	DMG4a	bar	DMG4b	block	5%
5	S10	*x* = 9 m~10 m	DMG5a	bar	DMG5b	block	5%
	S20	*x* = 19 m~20 m		bar		block	5%
6	S10	*x* = 9 m~10 m	DMG6a	bar	DMG6b	block	5%
	S20	*x* = 19 m~20 m		bar		block	10%
7	S10	*x* = 9 m~10 m	DMG7a	bar	DMG7b	block	5%
	S15	*x* = 14 m~15 m		bar		block	5%
8	S10	*x* = 9 m~10 m	DMG8a	bar	DMG8b	block	5%
	S15	*x* = 14 m~15 m		bar		block	5%
	S20	*x* = 19 m~20 m		bar		block	5%

**Table 3:** Specifications of wireless accelerometer.

Range (g)	±5
Sensitivity (mV/g)	400
Frequency Response (Hz)	0–600
Output Noise, Differential [Root Mean Square, typical] ($\mu g/\sqrt{\mathrm{Hz}}$)	7
Max Shock [(0.1 ms)] (g)	2000
Non-Linearity (±90%FSO)	0.5%
Bias Tem. Shift (T=−55 °C to +125 °C)	0.0001 g/°C
Type	Micro-machined capacitive sense element
Excitation Voltage (VDC)	+5.0 VDC (±0.025 VDC)
Output Impedance	50 Ω
Operating Current	15 mA @ 5.0 VDC
Differential Output	±2.0VDC FSO
Cross Axis Sensitivity	<=3%
Operating Temperature	−55 °C to +125 °C
Damping	Nitrogen Gas Damped
Overal Size (A×B×C)	49.5 mm×12.4 mm×20.3 mm
Weight (g)	54
Housing	6061 Aluminunum, IP67 rated
Cable	BDI-RC-187 (specify length)

**Table 4:** Stiffness change of partial unit of undamaged case.

Element Number	Unmodified EI	Modified EI	ΔEI
5	2.71 × 10^9^	2.09 × 10^9^	6.20 × 10^8^
7	2.71 × 10^9^	2.38 × 10^9^	3.33 × 10^8^
10	2.71 × 10^9^	2.21 × 10^9^	5.00 × 10^8^
13	2.71 × 10^9^	2.53 × 10^9^	1.80 × 10^8^
14	2.71 × 10^9^	2.49 × 10^9^	2.11 × 10^8^
17	2.71 × 10^9^	1.89 × 10^9^	8.25 × 10^8^
20	2.71 × 10^9^	2.07 × 10^9^	6.40 × 10^8^

**Table 5:** Stiffness change of partial unit of case 1a.

Element Number	Undamaged $EI_{u}$	Damaged $EI_{d}$	ΔEI	ΔEIEIu (%)
5	2.09 × 10^9^	2.03 × 10^9^	6.48 × 10^7^	0.031
7	2.38 × 10^9^	2.35 × 10^9^	3.33 × 10^7^	0.014
10	2.21 × 10^9^	1.73 × 10^9^	4.81 × 10^8^	0.218
13	2.53 × 10^9^	2.43 × 10^9^	9.87 × 10^7^	0.039
14	2.49 × 10^9^	2.44 × 10^9^	4.73 × 10^7^	0.019
17	1.89 × 10^9^	1.68 × 10^9^	2.08 × 10^7^	0.011
20	2.07 × 10^9^	1.98 × 10^9^	8.90 × 10^7^	0.043
